# Supplementary material for: Evaluation of the Immunosafety of Cucurbit[n]uril In Vivo
Source: Pharmaceutics. 2024 Jan 19;16(1):127. doi: 10.3390/pharmaceutics16010127 (PMC10820314; doi:10.3390/pharmaceutics16010127)
Supplement: Supplementary file 1 [file pharmaceutics-16-00127-s001.zip › pharmaceutics-2740298-supplementary.pdf]

Supplementary material for article

Evaluation of the Immunosafety of Cucurbit[n]uril In Vivo

Ekaterina Pashkina [1,2,*], Alina Aktanova [1,2], Olga Boeva [1], Maria Bykova [1], Elena Gavrilova [1], Elena Goiman [1], Ekaterina Kovalenko [3], Na'il Saleh [4], Lyubov Grishina [1] and Vladimir Kozlov [1]

[1] Research Institute of Fundamental and Clinical Immunology, 14, Yadrintsevskaya st., 630099 Novosibirsk, Russia
[2] Department of Clinical Immunology, Novosibirsk State Medical University, 52, Krasny Prospect, 630091 Novosibirsk, Russia
[3] Nikolaev Institute of Inorganic Chemistry, 630090 Novosibirsk, Russia; e.a.kovalenko@niic.nsc.ru
[4] Department of Chemistry, College of Science, United Arab Emirates University, Al Ain P.O. Box 15551, United Arab Emirates; n.saleh@uaeu.ac.ae
* Correspondence: eapashkina@niikim.ru

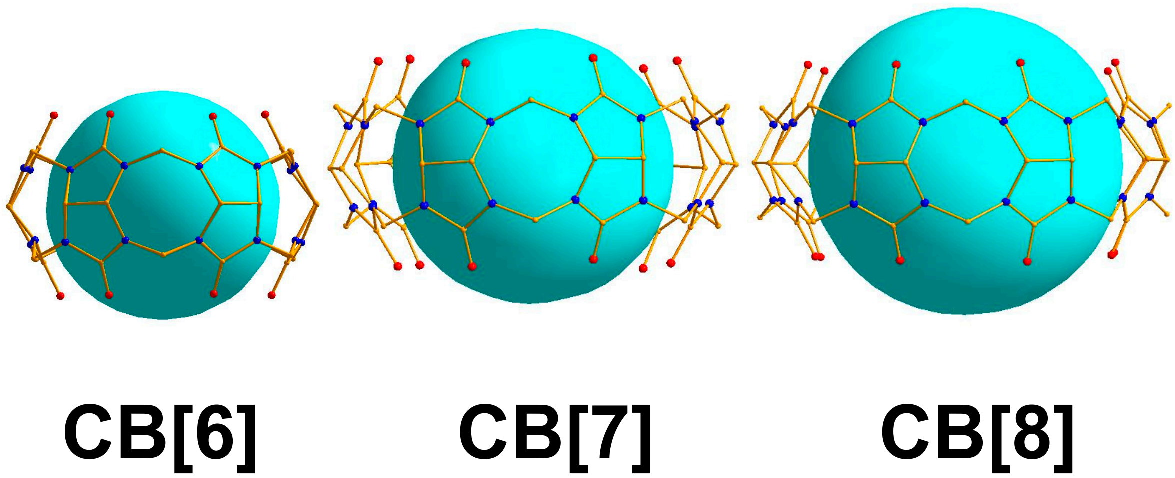

Figure S1. Cucurbit[n]urils (n=6,7,8; CB[6]·10H_2O, MW = 1177; CB[7]·10H_2O, MW = 1343; CB[8]·10H_2O, MW = 1509) structures and cavity size.

.

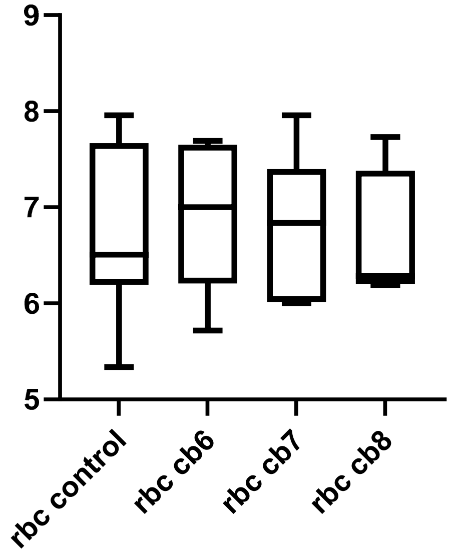

Figure S2. Effect of intraperitoneal administration of cucurbiturils on the number of red blood cells. Data are presented as box-and-whisker plots, with boxes extending from the 25th to the 75th percentile, with a horizontal line at the median, while the whiskers extend to the lowest and highest data points

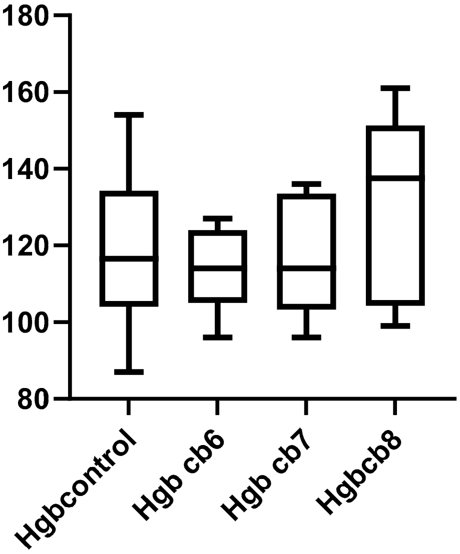

Figure S3. Effect of intraperitoneal administration of cucurbiturils on the hemoglobin level. Data are presented as box-and-whisker plots, with boxes extending from the 25th to the 75th percentile, with a horizontal line at the median, while the whiskers extend to the lowest and highest data points

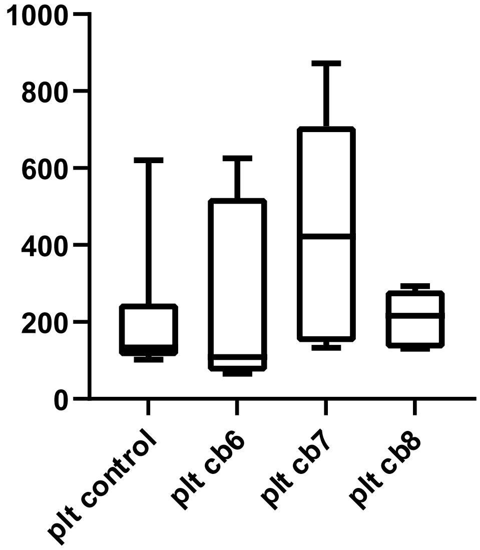

Figure S4. Effect of intraperitoneal administration of cucurbiturils on the number of platelets. Data are presented as box-and-whisker plots, with boxes extending from the 25th to the 75th percentile, with a horizontal line at the median, while the whiskers extend to the lowest and highest data points

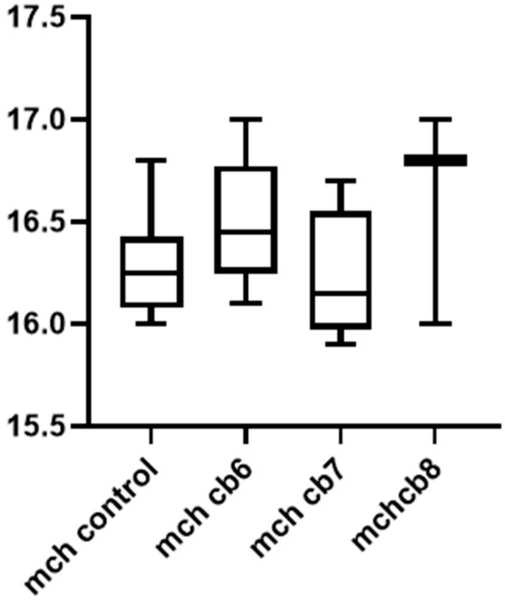

Figure S5. Effect of intraperitoneal administration of cucurbiturils on the mean corpuscular hemoglobin (MCH). Data are presented as box-and-whisker plots, with boxes extending from the 25th to the 75th percentile, with a horizontal line at the median, while the whiskers extend to the lowest and highest data points.

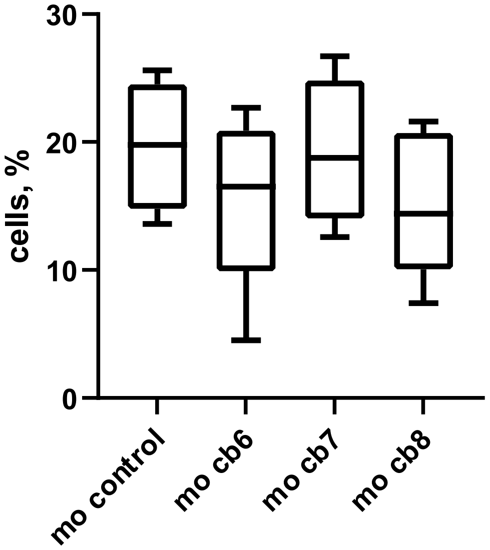

Figure S6. Effect of intraperitoneal administration of cucurbiturils on the number of monocytes. Data are presented as box-and-whisker plots, with boxes extending from the 25th to the 75th percentile, with a horizontal line at the median, while the whiskers extend to the lowest and highest data points

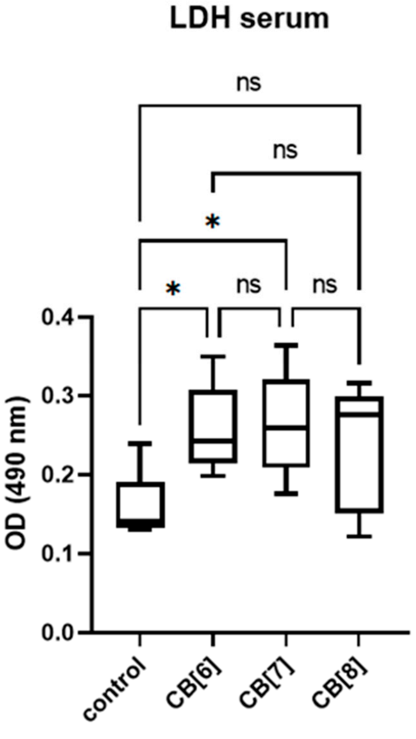

Figure S7. Lactate dehydrogenase (LDH) serum level after intraperitoneal cucurbituril administration. Data are presented as box-and-whisker plots, with boxes extending from the 25th to the 75th percentile, with a horizontal line at the median, while the whiskers extend to the lowest and highest data points. * Indicates a significant difference ($p < 0.05$) vs. the control.
